# The International Performance, Resilience and Efficiency Program Protocol for the Application of HRV Biofeedback in Applied Law Enforcement Settings

**DOI:** 10.1007/s10484-024-09644-3

**Published:** 2024-04-24

**Authors:** Judith P. Andersen, Joseph Arpaia, Harri Gustafsberg, Steve Poplawski, Paula M. Di Nota

**Affiliations:** 1 Department of Psychology, University of Toronto Mississauga, Toronto, Mississauga, ON Canada; 2 Affiliated Faculty, Temerty Faculty of Medicine, University of Toronto, Toronto, ON Canada; 3 Private Practice, Eugene, OR USA; 4 Police University College of Finland, Tampere, Pirkanmaa Finland; 5 Step Training Inc., Freelton, ON Canada

**Keywords:** Police, Autonomic modulation, Resilience, Heart rate variability biofeedback, Performance, iPREP

## Abstract

Law enforcement officers are routinely exposed to high-threat encounters that elicit physiological stress responses that impact health, performance, and safety. Therefore, self-regulation using evidence-based approaches is a priority in police research and practice. This paper describes a five-module heart rate variability biofeedback (HRVB) protocol that is part of a larger resilience program (the International Performance Resilience and Efficiency Program – iPREP) established in 2014. Supported by 10 years of user-informed research and development, our methods are tailored to address occupational stressors and the practical realities of training and resource availability in operational settings. Building on existing clinical methods that comprise five to six weekly sessions and up to 40-min of daily practice, our iPREP HRVB protocol is typically delivered in a condensed format across 2–3 days and is seamlessly integrated with reality-based training scenarios commonly employed in policing. By combining best practices in clinical HRVB with police-specific pedagogical frameworks, officers receive accelerated and job-relevant training to adaptively modulate autonomic responses to acute and chronic stress. Efficacy of the iPREP HRVB protocol is supported by several research studies of various methodological designs (i.e., randomized control trial, longitudinal cohort) that demonstrate immediate and sustained improvements in police performance and physiological health outcomes. We conclude with a critical appraisal of the available empirical evidence contrasting common and emerging breathing techniques proposed for use in operational policing contexts. The critical appraisal guide is intended to serve as a resource for law enforcement agencies, governing bodies, and operators when choosing appropriate and effective self-regulation training approaches.

**Supplementary Information:**

The online version contains supplementary material available at 10.1007/s10484-024-09644-3.

## Introduction

This paper presents our heart rate variability biofeedback (HRVB) protocol used in research and applied practice with law enforcement officers including police constables and tactical emergency response workers (i.e., special intervention units or SWAT teams). The HRVB protocol is part of a larger and more comprehensive police training curriculum called the International Performance Resilience and Efficiency Program (iPREP), which is currently implemented at national police colleges and organizational training centers in North America and Europe (Andersen et al., 2021; Di Nota et al., 2021a, b, c). Our HRVB method has been refined from established clinical protocols that require 5–6 one-hour weekly sessions in addition to recommended practice (~ 20 min/day) (Lehrer et al., 2013). Significant benefits have been reported for a wide variety of physical and mental conditions following clinical interventions that follow this approximately 12 h of HRVB training and practice (for systematic review and meta-analysis see Lehrer et al., 2020). In contrast, the current HRVB protocol involves approximately 24 h of HRVB training and practice, which is typically delivered over the course of two to three days (see Table S1 in Supplementary Materials for sample schedule). The integration of HRVB into contemporary active police practices, including scenario-based training, accelerates job-relevant learning for officers while addressing the realities of limited resource availability for non-essential training (i.e., not mandated by individual police agencies or organizations). In this paper we refer to ‘modules’ rather than sessions to better clarify the order in which we teach iPREP HRVB concepts to address short- and long-term stress and integrate these techniques with reality based (scenario) training. Our evidence-based protocol has been refined over a decade of multi-method applied research studies with law enforcement officers and has been used in private clinical practice for over 20 years by the second author (Andersen et al., 2021).

A systematic review of interventions designed to mitigate the impact of occupational stressors among public safety personnel (PSP) including police, reveals that programs integrating HRVB were the only interventions showing effectiveness in improving physiological processes (modulating heart rate (HR) during high-threat reality-based scenarios) (Di Nota et al., 2021a). The lack of efficacy of other intervention types (e.g., mindfulness, general wellness or resilience training) is most likely because they do not address the underlying dysregulations in physiology associated with unease in occupational contexts (Andersen et al., 2023; Arpaia & Andersen, 2019). The methods employed in the iPREP HRVB protocol are built from established protocols (Lehrer et al., 2013) and tailored for law enforcement. Studies support the efficacy of the protocol in improving performance and health immediately post intervention and at long term follow up (Andersen & Gustafsberg, 2016; Andersen et al., 2015; 2016a, b; 2018a, b). Importantly, empirical evidence indicates that our protocol should not be shortened to a one-day session, despite interest to do so by law enforcement administrators (Di Nota et al., 2021b).

Threatening, uncertain or potentially conflictual interactions increase unease, a state governed by internal physiological processes that direct cognition and behavior (Andersen et al., 2020; Arpaia & Andersen, 2019). Police training in North America focuses primarily on external factors (e.g., time, tools) and technical skills (e.g., weapons and tactics) and lacks standardized education on stress awareness and modulation (Huey et al., 2021; Bennell, Alpert, Andersen et al., 2021). Notably, police training often fails to provide officers with skill-based learning opportunities (e.g., reality-based training (RBT) scenarios) that specifically train them to recognize and effectively modulate internal physiological responses to stress despite knowing that these responses exist in both training and field duty (Baldwin et al., 2019, 2022). Unease is not ameliorated by telling officers to ‘calm down’ ‘show no weakness, or ‘embrace the suck’, which are commonplace directives in police culture and training (Rawski & Workman-Stark, 2018). The good news is that HRVB addresses the above gap in police training along with many additional benefits, including being non-invasive, low cost, straightforward to administer and has been met with high buy-in from police.

**Fig. 1:**
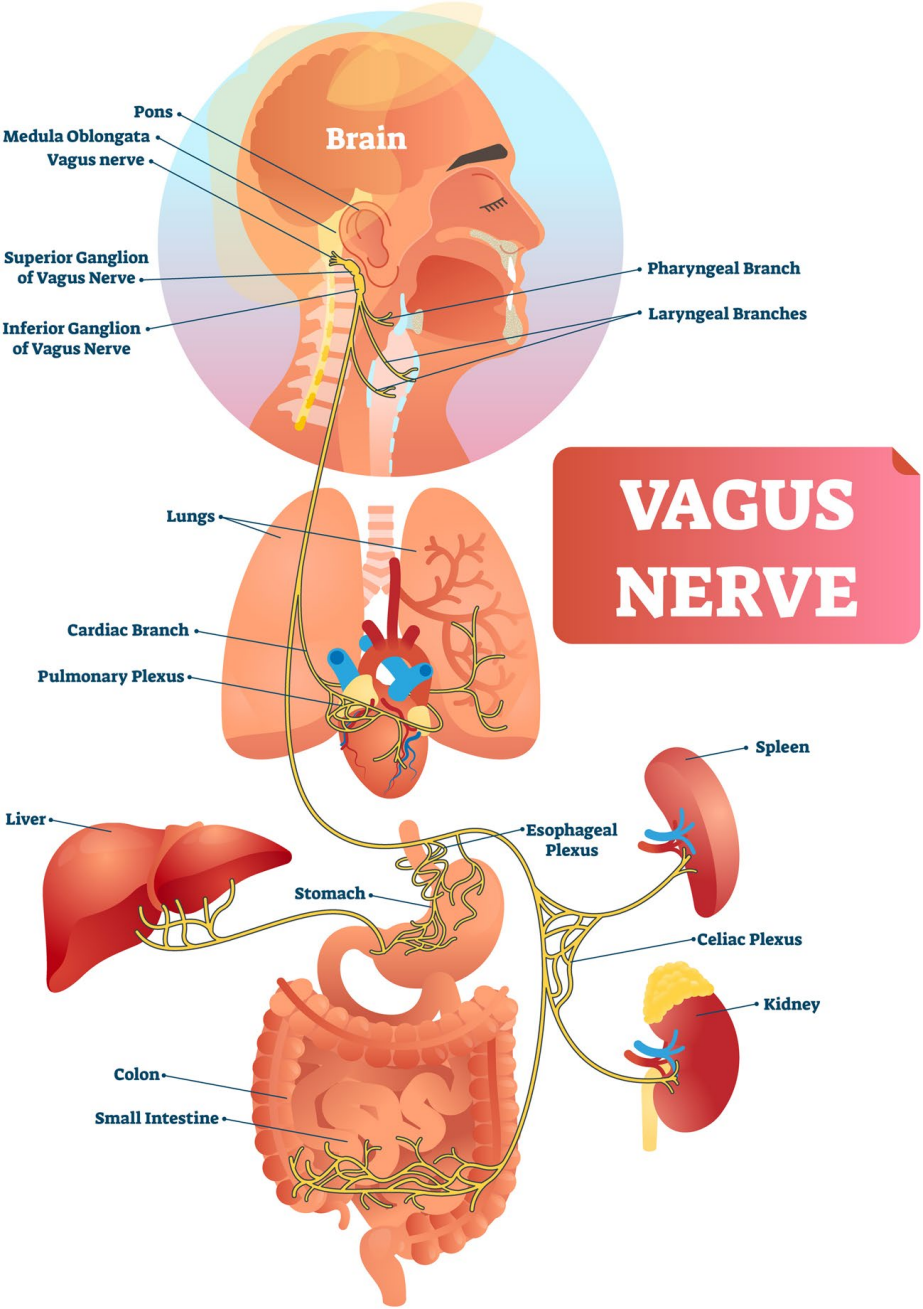
The vagus nerve. The vagus nerve innervates key organ systems. Photo provided by J.P. Andersen (Adobe Stock #254408845)

The efficacy and methodology of HRVB in clinical settings is described in detail elsewhere (Lehrer et al., 2013; Vaschillo et al., 2006). Briefly, these methods involve training a form of paced breathing at a ‘resonance frequency’ (RF) in line with the cardiovascular system. Breathing in this manner causes HR to fluctuate in phase with respiration (i.e., HR increases with inhalation, decreases with exhalation). The specific pace or resonance frequency varies across individuals and is identified by the following characteristics: a) observing a smooth sinusoidal HR trace that is in phase with respiration; b) a high peak-to-trough amplitude; and c) high low frequency HRV peak amplitude. Short-term benefits of paced breathing include stimulation of the baroreflex, maximizing gains in respiratory sinus arrythmia and gas exchange efficiency (Vaschillo et al., 2006; Yasuma & Hayano, 2004). Regular practice of RF breathing has resulted in clinically significant improvements in emotional processing, physical and mental health, (e.g., anxiety, depression, anger), athletic and artistic performance, and cognitive performance (e.g., attentional control, working memory) (see Lehrer et al., 2020 for meta-analysis). The mechanism by which RF, and indeed the current iPREP HRVB protocol, improves cognitive and physiological functioning in resting, reactivity, and recovery states are explained by various established theories (vagal tank theory: Laborde et al., 2018; polyvagal theory: Porges, 2007; neurovisceral integration model: Thayer et al., 2009). Clearly, modulation of the vagus nerve (see Fig. 1), and the principal organs it innervates, has profound implications for directing behavior, cognition and health.

**Fig. 2:**
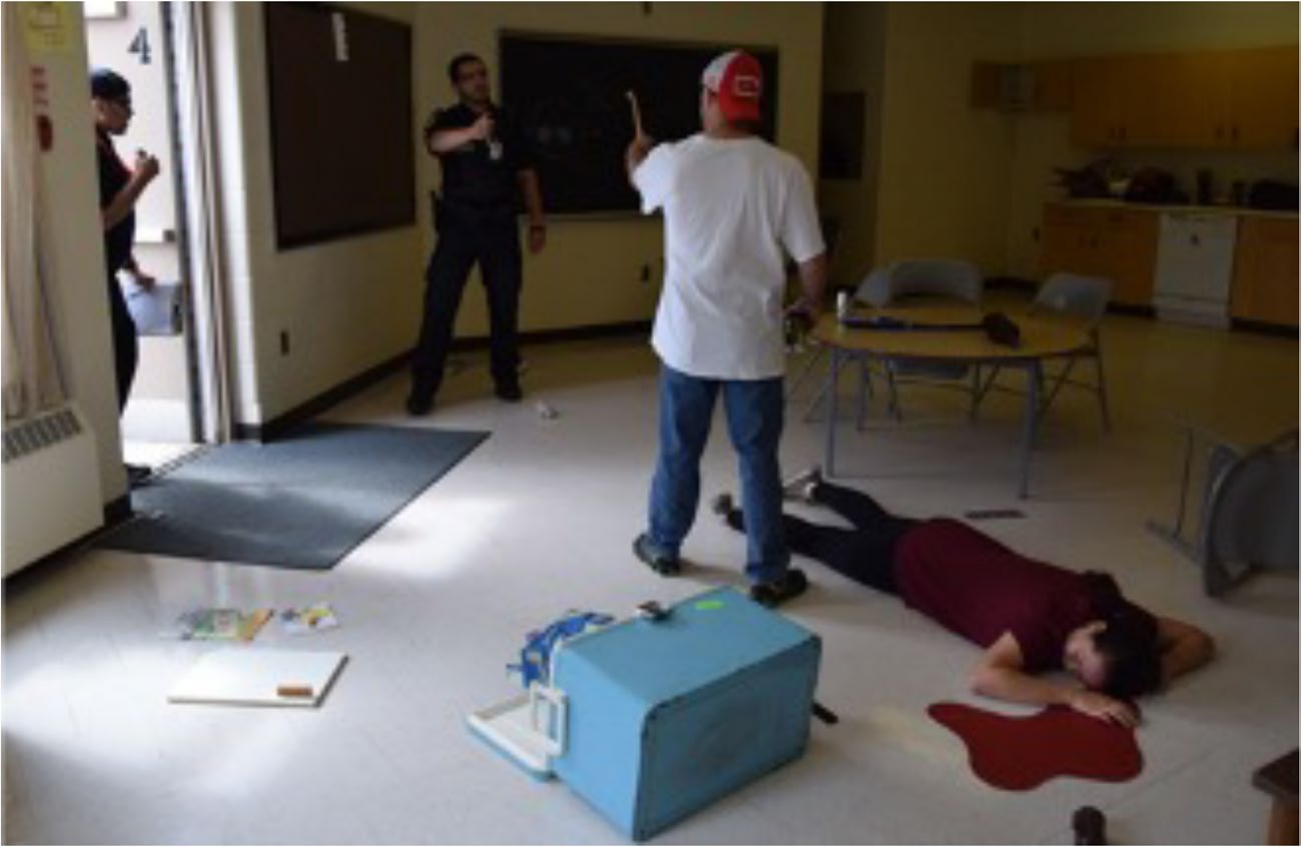
Example of a law enforcement reality-based training (RBT) exercise. RBT uses props and infrastructure to stage various environments (e.g., apartment, rooming house) in dedicated training rooms at individual agencies or at real locations (e.g., decommissioned schools or buildings). Experienced police instructors or trained actors deliver scripted behaviors that are designed to elicit, train, or evaluate specific skills (i.e., de-escalation, verbal communication, positioning, physical maneuvers, critical decision-making) based on predetermined outcomes (e.g., subject will comply with officer commands; subject will not comply, requiring a use of force). Image copyrighted to Judith P. Andersen

The development of the iPREP HRVB protocol for field application among law enforcement officers is informed by the collective expertise of the co-authors, comprised of health and cognitive psychologists, clinicians well-versed in administering HRVB protocols, and experienced police operators and instructors. Building on extant methods and technology, the iPREP HRVB protocol delivers **four novel contributions** that utilize brief and extended periods of HRVB training in active law enforcement contexts:

A. ***Reset, Refocus, Respond (RRR):*** brief HRVB practice to visualize the adaptive modulation of respiratory sinus arrhythmia (RSA), the synchrony between heart rate and breathing, during acute stress (Andersen et al., 2023). The RRR technique facilitates the shift of attention away from physiological unease and back onto ‘what’s important now.’ Prolonged exhalation is more effective at promoting performance during a stressful task compared to other breathing techniques that can pose safety risks by depleting cognitive and physiological resources (see Table 1).
B. ***Recovery Breathing:*** the use of extended HRVB practice to manage chronic stress and build reserves. Adapted from Lehrer et al.’s (2013) clinically validated RF protocol, officers are assisted in identifying the breathing pace that visually approximates their RF (the synchrony between HR and respiration) using HRVB methods in the field. Our HRVB protocol also emphasizes that the RF pace must be comfortable. Participant comfort facilitates conditioning and boosts adherence to practicing the technique, a factor that is rarely emphasized in existing breathing protocols or studies (Balban et al., 2023; Steffen et al., 2017; Vaschillo et al., 2006).
C. ***Field technology:*** development and use of technology and methods that both: a) address limitations of existing HRVB approaches including comfort, which promotes continued practice, and b) are tailored for integration into operational police settings. Our approach presents raw real-time HR beat-to-beat intervals with no time delay and superimposed on a video recording of officer scenario performance, increasing credibility and buy-in. HRVB is presented using ambulatory heart monitors paired with a tailored app that is secure (not cloud based), easily interpretable by non-clinicians, and highly acceptable to end users (i.e., face validity) (see section on ‘Technological developments for HRVB in field settings’). Importantly, we present raw beat-to-beat HR data during iPREP HRVB because it represents the neural activation or inhibition of the autonomic nervous system in real time (Billman, 2013). In contrast, commercially available products or programs typically present HRV algorithms or a general ‘stress’ or ‘coherence’ index generated from aggregated signals of autonomic activity that do not provide valid or interpretable data for modulating physiology in real-time ambulatory contexts.
D. ***Integration of HRVB with occupationally relevant training:*** iPREP HRVB skills are conditioned during reality-based training (RBT) scenarios that are: a) representative of officers’ field duties (Fig. 2) and b) can be seamlessly and conveniently integrated with existing RBT practices and agency resources.

**Table 1:** Common breathing techniques employed in law enforcement settings. The following summarizes the benefits and limitations of various breathing techniques commonly employed for improving health, functioning, and/or performance in police and law enforcements settings, including training and active duty. While not a comprehensive list, we have focused on summarizing extant empirical literature on breathing techniques commonly utilized in active settings (i.e., not for the purpose of relaxation or meditation). To critically appraise the listed sources, strength of evidence is noted and based on two criteria: 1) findings in support of the technique (i.e., demonstrating adaptive or advantageous effects) published in a peer-reviewed empirical source (e.g., journal article); 2) findings based on applied police, public safety, or military sample

Breathing technique	Description	Study	Sample *n* (Country)	Benefits	Limitations	Strength of evidence
*Single breath*						
Reset Breath/One Breath	Deep inhalation through the nose, brief 1 s hold, prolonged exhalation through pursed lips	Andersen & Gustafsberg, 2016;Andersen et al., 2015; 2016a, b; 2018a, b	2015: 18 tactical officers; 2016: 12 tactical officers (6 experimental, 6 control) (Finland); 2018: 57 police officers (Canada)	Immediate HRVB intervention to modulate physiological responses to acute stressors; has been integrated into scenario based training and active duty complex tasks. The technique does not require deliberate thought processes to promote psychophysiological preparedness, performance, and recovery	Terminology has evolved over time, but the technique remains unchanged.	◆◆
Sighing/Physiological sigh/Prolonged exhalation	One or two deep, fast inhalations with prolonged exhalation that together are 2–4 times larger than preceding and following breaths	Psychophysiological reset model by Vlemincx et al. (2013); Röttger et al., 2021	30 officer cadets and civilian students (15 box/tactical breathing, 15 prolonged exhalation at 2:8 phase ratio) (Germany)	Immediate reset of respiratory variability and emotional regulation; can be purposefully or involuntarily initiated in response to sensory stimuli or emotional states; improves performance accuracy and reaction time relative to box/tactical breathing	Excessive or continuously repeated sighing may lead to respiratory instability and subjective feelings of discomfort	◆◆
*Prolonged paced breathing*						
Box breathing/ tactical breathing/combat breathing	Consistent timing (typically 4-5 s) of inhalation, holding, exhalation, holding	Röttger et al., 2021; popularized by Grossman (2014)	30 officer cadets and civilian students (15 box/tactical breathing, 15 prolonged exhalation at 2:8 phase ratio) (Germany)	Tactical breathing results in lower HR and respiratory rate during and following a Stroop task but performance accuracy and reaction time are less effective compared to prolonged exhalation	Counting breaths requires cognitive resources and is distracting during active duty contexts; holding one’s breath limits gas exchange and can lead to hyperventilation	◆
Focused breathing	Unstructured, deep, slow, consistent abdominal breathing with focused attention inward or outward	Hourani et al., 2011; Lewis et al., 2015	77 Marines (43 experimental, 34 control) (USA); 891 military personnel pre-deployment (469 experimental, 422 control) (USA)	Improved physiological functioning in virtual combat training scenarios; distinct techniques for in-combat attentional processes and post-combat recuperation	Recuperative technique is done with eyes closed, limiting use in active duty contexts	◆◆
Prescribed paced breathing; Freeze Frame	Assignment of prescribed breathing pace (typically 5-6 bpm) of inhalation and exhalation, typically guided by visual or auditory cues (e.g., HeartMath)	Andersen & Gustafsberg, 2016;Andersen et al., 2015; 2016a, b; McCraty & Atkinson, 2012	2016: 12 Tactical officers (6 experimental, 6 control) (Finland); 2015, 2016 & 2016a: 18 tactical officers (Finland); 2012: 59 police officers and city manager (28 experimental, 31 control) (USA)	HRVB training taught in a classroom and adapted for applied field settings (critical incident scenarios); improved post-training average HR, instructor ratings on focus, decision-making, and communication, and self-reported satisfaction and emotion regulation	One prescribed breathing pace for all individuals may lead to subjective feelings of discomfort for people with a different resonance frequency (see below)	◆◆
Cyclic Sigh	Repeated (*M* = 6 min) practice of cyclic sigh (two inhalations through the nose, prolonged exhalation through the mouth) for *M* = 20 days	Balban et al., 2023	108 healthy adults (30 cyclic sigh, 21 box breathing, 33 cyclic hyperventilation, 24 mindful meditation) (USA)	Improved self-reported mood and reduced respiratory rate during sleep when compared to daily 5 min practice for 1 month of box breathing and cyclic hyperventilation (longer inhalation, shorter exhalation)	Not empirically validated using in-person protocol or in active, applied law enforcement settings; no evidence for improvements in cognitive functioning or performance	◆
Resonance frequency (RF) breathing^2^	Paced breathing at a preferred individualized frequency that optimizes HRV and synchronizes cardiorespiratory phases	Steffen et al., 2017	95 healthy undergraduate students (RF, RF + 1 breath/min, control groups) (USA)	Empirically validated technique for clinical populations and undergraduates; improved physiological functioning at rest, during stressful tasks, and following stress	Not empirically validated in applied law enforcement settings	◆
Recovery breathing	Paced breathing at a preferred individualized frequency that optimizes HRV, synchronizes cardiorespiratory phases, and subjectively feels comfortable	Andersen et al., 2018a, 2018b	57 police officers (Canada)	Empirically validated; HRVB training adapted for applied field settings; finds their optimal breathing pace through a fine-tuned process tailored to the individual’s physiology (0.2 breaths/min)	Requires multi-day training with HRVB integrated into, and practiced after, live reality-based scenarios	◆◆

^2^For the numerous clinical benefits, see the meta-analysis of findings on HRVB-trained resonance frequency breathing Lehrer et al. (2020). None of the studies included in the meta-analysis investigated HRVB in police, law enforcement, or military samples

## Manual for HRVB Training in Field Settings with Law Enforcement Officers

- Module 1: Background and introduction to stress physiology and HRVB
- Module 2: Intervention for acute stress: Reset, Refocus, Respond (RRR)
- Module 3: Practice RRR using HRVB and RBT
- Module 4: Intervention for chronic stress: Recovery Breathing
- Module 5: Integrating HRVB practice for RRR and Recovery Breathing using RBT

## Module 1: Background and introduction to stress physiology and HRVB

**Fig. 3:**
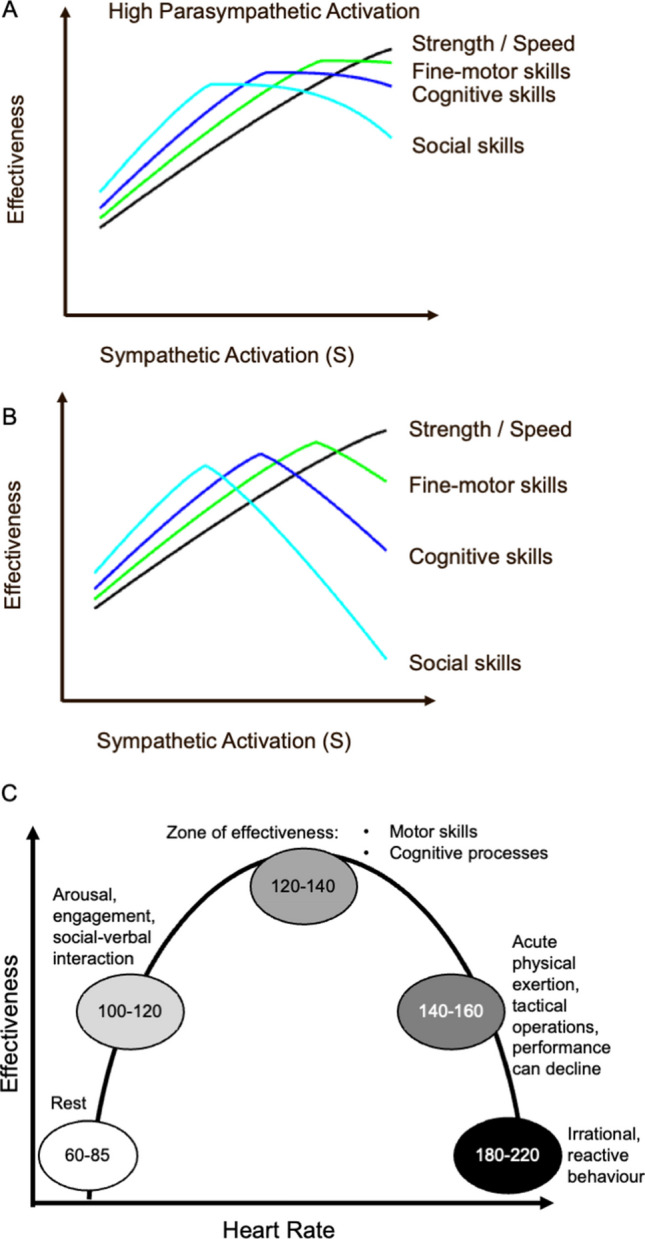
Physiological arousal and performance. **a** The combination of high sympathetic (SNS) and parasympathetic nervous system activation is an adaptive physiological state that supports effective performance in physical/motoric, cognitive, and social domains. **b** While high SNS activation is adaptive for purely physical activities, such as running or lifting, it is not adaptive for sustained performance of cognitive, social or fine motor skills. **c** Heart rate arousal curve, based on the Yerkes Dodson model (1908), accumulated evidence from applied research (Di Nota & Huhta, 2019) and the co-authors collective experience in clinical, applied, and psychophysiological research

The content covered in Module 1 is intended to frame HRVB as immediately relevant to officers’ operational job contexts. Officer buy in or belief in the effectiveness of the protocol is increased from the very start of the program through an overview of stress physiology that is consistent with the research literature but put forward in concrete terms with minimal scientific jargon. Reviewing the applied psychophysiological literature in police and law enforcement contexts facilitates further credibility of and motivation to learn the physiological self-regulation techniques (Di Nota & Huhta, 2019; Di Nota et al., 2021a, b, c). Instructors paraphrase the following:

1. Introduce instructors, describe the five-module program and enact agency training safety protocols (i.e., physical check to ensure that all duty weapons have been deactivated or replaced with training equipment as is common practice in law enforcement).
2. Emphasize that HRVB is an evidence-based intervention that is rooted in adult learning theory (Andersen et al., 2017; Di Nota & Huhta, 2019; Di Nota et al., 2021a, b, c), clinical practice (Lehrer et al., 2013, 2020), and has established effectiveness in improving performance and health outcomes in numerous law enforcement samples (Andersen & Gustafsberg, 2016; Andersen et al., 2015; 2016a, b; 2018a, b; McCraty et al., 2009; McCraty & Atkinson, 2012).
3. Review adaptive and non-adaptive stress physiology from basic science and law enforcement perspectives.
  - Adaptive response to stress (Lovallo, 2015) (Fig. 3a)
    i. Improved cognitive, behavioral and sensory/perceptual outcomes.
    ii. HR and oxygen increase in response to chemical cascade (catecholamines, adrenalin).
    iii. Autonomic nervous system (ANS) is fully engaged (dynamic interplay between sympathetic (SNS) and parasympathetic (PNS) branches).
    iv. Adequate hypothalamic pituitary axis (HPA) axis response.
  - Non-adaptive response to stress (Fig. 3b)
    i. As relevant to police contexts, ANS over or under activation results in a variety of detriments, including cognitive, sensory and perceptual narrowing that may hinder performance during field duties (e.g., poor motor control, reduced situational awareness, learning and memory) (Anderson et al., 2019; Baldwin et al., 2022; Di Nota & Huhta, 2019). Optional training exercises include using examples of non-adaptive stress physiology in police contexts (e.g., publicly available videos of police behaviour that demonstrate officers clearly showing signs of stress and responding poorly to calls for service (e.g., chaotic movements; stuck in the same command loop; vocal dysregulation including high, yelling, or angry tone; heavy breathing or hyperventilation; shaking; excessive sweating; incorrect behavioral responses). Engage group discussion to identify the signs of stress and how they impact operational decision-making and performance. Observing officers in action draws in the officers’ attention, facilitates participation, and helps them link stress physiology with occupational outcomes through an observational learning exercise.
4. Describe that there are no exact HR values that directly lead to adaptive versus non-adaptive performance, but rather, research has demonstrated general zones of effectiveness (Fig. 3c). During this training each individual will identify their own zone at which they are most effective. Effectiveness zones can be determined by observing the individual perform several scenario-based exercises while monitoring their HR and behavior. Importantly, the objective HR indicators of stress ***must*** be combined with direct behavioral observation to provide context for interpreting *meaningful* and *valid* changes in physiological activity. HR changes observed without knowing the context in which they occurred cannot be interpreted accurately.

### Module 2: Intervention for Acute Stress: Reset, Refocus, Respond (RRR)

Allow participants a 15-min rest break between Module 1 and this next component (Module 2). The short break allows for a cognitive attentional rest and students will be better able to absorb the following information. The purpose of Module 2 is to present HRVB as one tool that assists the officer in modulating ANS activation to improve performance and reduce the likelihood of accumulating the health risks associated with repeated stress exposures (i.e., allostatic load over time, McEwen, 1998).

The **Reset, Refocus, Respond (RRR)** technique is initially trained in a classroom setting. The next module will integrate RRR into reality-based training (RBT) scenarios using HRVB. The biological structure and mechanisms by which the Reset Breath is beneficial to optimize autonomic modulation during acute stress is described elsewhere in detail (see Andersen et al., 2021; Andersen et al., 2023). For simplicity’s sake, we do not go into great detail on the biological or physiological mechanisms when training officers. Rather, we teach the skills at a level and in a manner that enhances skill retention and application in occupationally relevant contexts according to adult learning practices (Di Nota & Huhta, 2019; Di Nota et al., 2021a, b, c; Jenkins et al., 2020; Körner & Staller, 2020).

**Fig. 4:**
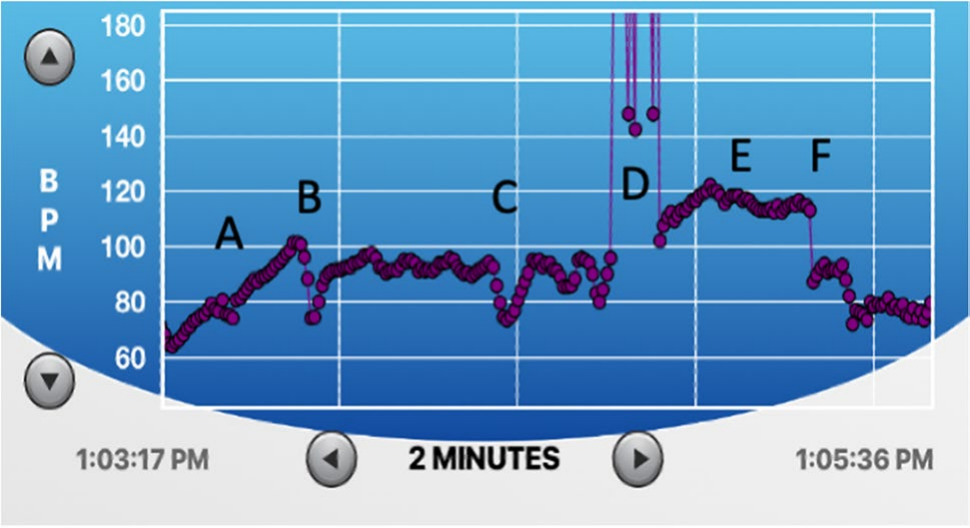
The Reset Breath in action. This is a screenshot of the HRV Trace app showing a 2-min portion of continuous heart rate (HR) data obtained from a physician during a live-action scenario (see ‘Technological developments for HRVB in field settings’ section for further details). Each purple dot represents a single heartbeat plotted continuously over time (x axis) in beats per minute (BPM, plotted on y axis). **A** Anticipatory stress increases HR from approximately 65 to 100 BPM as the physician waits to enter the scenario room. **B** First Reset Breath is performed to manually decrease HR and return to an adaptive physiological state with a refocus on the patient. **C** Second and third Reset Breaths performed to maintain adaptive physiological state. **D** Patient physically attacks physician and HR monitor is disrupted. **E** HR remains elevated due to the recent physical activity of self-defense, increasing oxygen demands on the body. **F** Acute physiological recovery is achieved by engaging in two successive Reset Breaths, returning the physician’s HR and physiological state to near pre-scenario levels upon completion of the scenario. Data image provided by J.P. Andersen

**Fig. 5:**
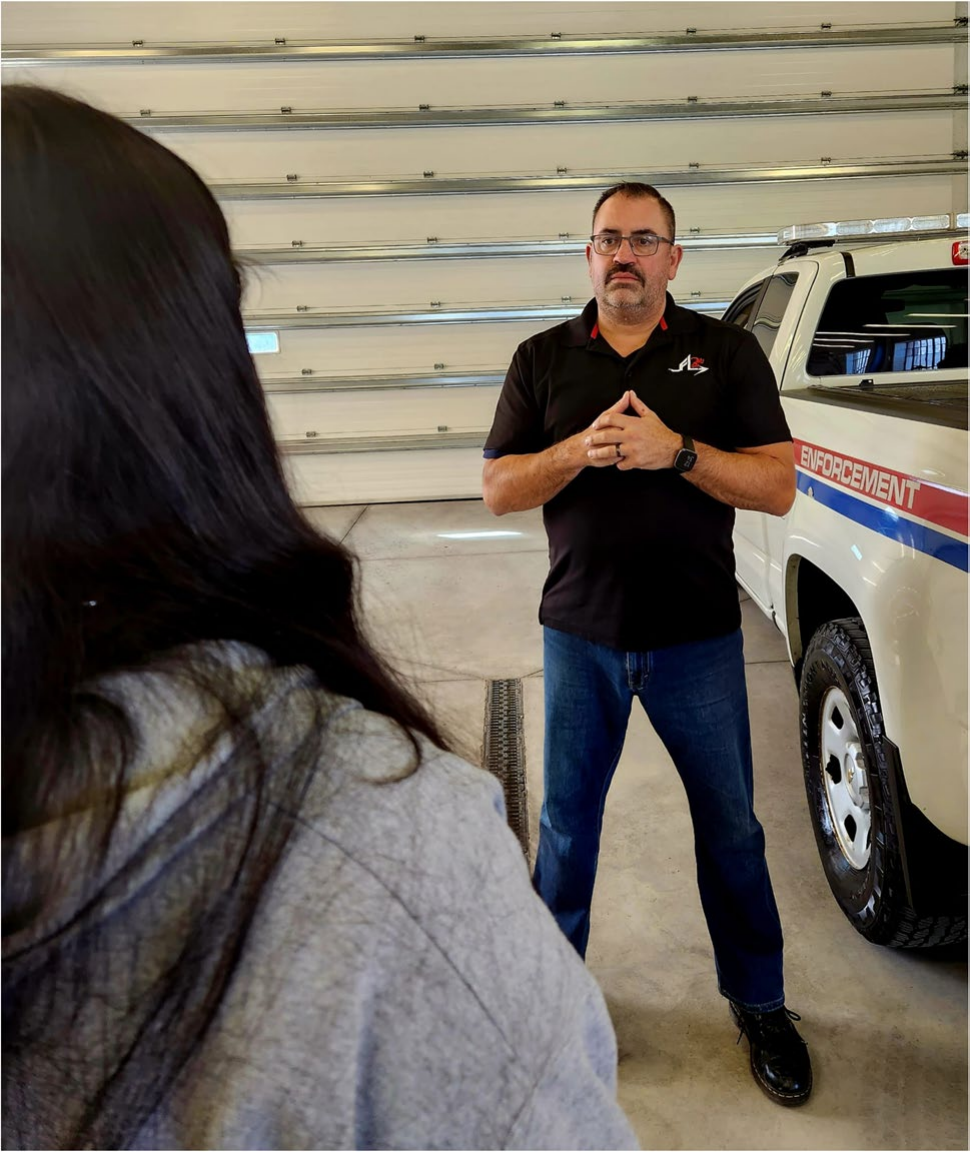
Sample image of police interview stance. The modelled body position provides several physiological and tactical advantages, including postural stability and improved groundedness, directed gaze and attention towards central cues, and increased readiness to mobilize for action. Image provided by S. Poplawski

1. The first technique instructed in our protocol is the ‘Reset Breath.’ Similar to a physiological sigh (Vlemincx et al., 2013), the Reset Breath stimulates the vagus nerve and signals the brain (see Fig. 1) to provide a short window during which the officer can shift attention from the unease that accompanies the stress response and refocus on the task at hand (Arpaia & Andersen, 2019; Bennell et al., 2021; Laborde et al., 2018; Vlemincx et al., 2013; Zelano et al., 2016). We teach officers that the Reset Breath is a physical ‘manual override’ that does not require cognitive control or (re)appraisal to provide immediate benefits (i.e., autonomic regulation and maintaining effectiveness). Rather, we can behaviorally condition the Reset Breath to be performed without having to consciously think about it. By implicitly engaging in this natural breathwork, it will elicit adaptive neurophysiological effects. To demonstrate the Reset Breath in action, show the sample HRVB screen trace (Fig. 4) and describe the following:
  - “*The graphic shown here depicts the real time HR response of a physician responding to a call to visit a patient’s room. In this scenario, the physician was told that when they enter the room, they will encounter a person that may engage in violent behavior. As you can see, during the first few seconds while the physician is waiting outside the patient’s room (marked with the letter ‘A’), the physician’s HR is escalating quickly from 60 to 100 BMP, demonstrating anticipatory stress. The physician knows that his HR level is typically between 80–100 and so implements the Reset Breath (marked ‘B’) to maintain his optimal level of autonomic reactivity (‘zone of effectiveness’). The physician then walks into the room, performs a series of Reset Breaths to maintain an adaptive state (marked ‘C’) before experiencing a violent reaction at point ‘D’. Subsequently, HR remains higher at point ‘E’ due to the recent physical exertion of defending themselves and increased oxygen demands on the body. At point ‘F’, the physician engages several Reset Breaths and rapidly returns to an adaptive physiological state.” The rapid return to the zone of effectiveness means that the physician will be able to successfully meet any subsequent challenges that might arise during the encounter. The ability to recover rapidly within a call is particularly relevant to law enforcement officers that may face many separate challenges within one incident.*
  - Discuss the operational and health-related benefits of being able to:
    i. Maintain an adaptive physiological state during a scenario or stressful operational task;
    ii. Return to an adaptive state following a surprise event during a call. The ability to modulate very quickly means that an officer will be prepared to deal with multiple unexpected crises within a call and recover physical and mental functioning; and
    iii. Return to an adaptive state following a scenario or call to be ready for the next one while maintaining energy reserves and reducing long-term wear and tear on the body.
2. Deliberate practice of the Reset Breath: Officers are instructed to stand and perform the Reset Breath several times according to the following script:
  - “You will now have an opportunity to physically practice the Reset Breath for the first time. Note that you will do this in an exaggerated manner for now but following scenario-based practice later today and in the coming days, you will learn to do the Reset Breath in a way that no one will be able to tell you are doing it in the field. Please stand where you are seated, and ensure your feet are spread shoulder width apart. I will first explain what we are going to do before we try it together a couple of times. First, you will fully exhale, then take a deep breath in through your nose. Be sure to fill your lungs as completely as possible – from top to bottom, keep your shoulders down and relaxed, chest open. Once you have taken a full breath, hold or pause briefly (~ one second) before exhaling **through pursed lips,** causing resistance as you exhale. Once you have exhaled completely, continue to breathe as you normally would.”
  - “Now let’s try it together. Begin to exhale completely… [wait approximately 2–3 s]… now inhale deeply through your nose, keeping your shoulders down, filling your lungs completely from top to bottom… [wait approximately 4- 5 s while students inhale]… now hold for one second at the top of your breath, and begin to exhale through pursed lips. Because we are just learning this technique, you can even puff out your cheeks for now to exaggerate a slow exhale. It is often helpful to feel as though you are physically pushing down through your core with your exhale, down to your feet, increasing a sense of groundedness, and manually driving your heart rate down. By inducing some resistance as you exhale and tightening your core and abdominal muscles, we are activating your diaphragm and vagus nerve that is directly connected to your heart, brain, lungs and other major organs, manually decreasing your stress response. This will allow you to Refocus your attention on what’s important now, broaden your situational awareness, and enable more effective Responding through better decision-making and motor control.”
  - As a major practical component of RRR training we have officers partner up and practice the Reset Breath while assuming a grounded position, often called the interview stance in policing (Fig. 5) for approximately 3 min (at least two Reset Breaths per partner). Make sure they and their partner are exaggerating the exhale at first. We also demonstrate how the Reset Breath can be done with a more subtle and covert exhalation when you are directly interacting with a person (i.e., after asking a question you can perform a Reset while they are responding).
  - Have officers practice the Reset Breath sitting down, which they can employ when seated in their patrol car or in an office setting. The same principles and techniques apply as when standing. Practicing the Reset Breath in a variety of contexts helps to encode and generalize the skills without deliberate or conscious effort.
  - Have officers partner up and practice the Reset Breath while seated for 3 min (approximately two Reset Breaths per partner).
3. The Refocus component of the RRR technique involves a ‘grounding’ procedure that facilitates a sense of embodiment and awareness of one’s internal physiological states, which is often overlooked or not trained at all (Andersen et al., 2017, 2018a; Di Nota et al., 2021a, b, c). A grounded state is in line with the interview stance, a body position that enhances safety and readiness for optimal physical action (see Fig. 5). Refocusing is also tied to increasing officers’ situational awareness, which is a critical policing skill that informs other important functions including visuomotor control, prediction of situational outcomes, and critical decision-making and has been defined in police-specific contexts elsewhere (Di Nota & Huhta, 2019; Di Nota et al., 2021a, b, c; Huhta et al., 2022, 2023). Refocusing attention to the critical aspects of the current moment provides officers with an important tactical advantage when responding under high-threat conditions; by training the anticipation (i.e., mental simulation) of alternative outcomes and corresponding responses, a person’s responses (i.e., decision-making and motor control) become faster and more accurate (Colin et al., 2014; Di Nota & Huhta, 2019). Thus, the RRR technique conditions autonomic modulation skills that broaden situational awareness, improve performance in acutely stressful situations, and improve self-regulation of physiological stress responses (Andersen & Gustafsberg, 2016; Andersen et al., 2015, 2016a, b, 2018a, b).
4. The third R in the RRR action for acute stress is ‘Respond’; by this we mean that the officer takes advantage of the window of clarity and autonomic modulation provided by the Reset Breath and the cognitive Refocus in order to Respond effectively to ‘what’s important now’, resolving the situation at hand (Willis, 2011).

### Module 3: Practice RRR using HRVB and RBT

This module teaches individuals how to integrate HRVB into RBT to 1) visually examine real-time HR fluctuations in synchrony with the officer’s observed behavior, and 2) confirm that officers are employing the RRR technique in occupationally relevant contexts. This method also affords law enforcement instructors a window into the officer’s internal physiological state so that they can provide objective, evidence-informed feedback. Specifically, instructors can intervene if they observe that officers are at risk of becoming physiologically overactivated (Fig. 3c) or confirm that officers are applying the RRR technique effectively based on the HRVB trace. For brevity we do not detail the design and delivery of critical incident RBT for law enforcement, but instead we refer readers to Jenkins et al. (2020) and Di Nota et al., (2021a, 2021b, 2021c).

**Fig. 6:**
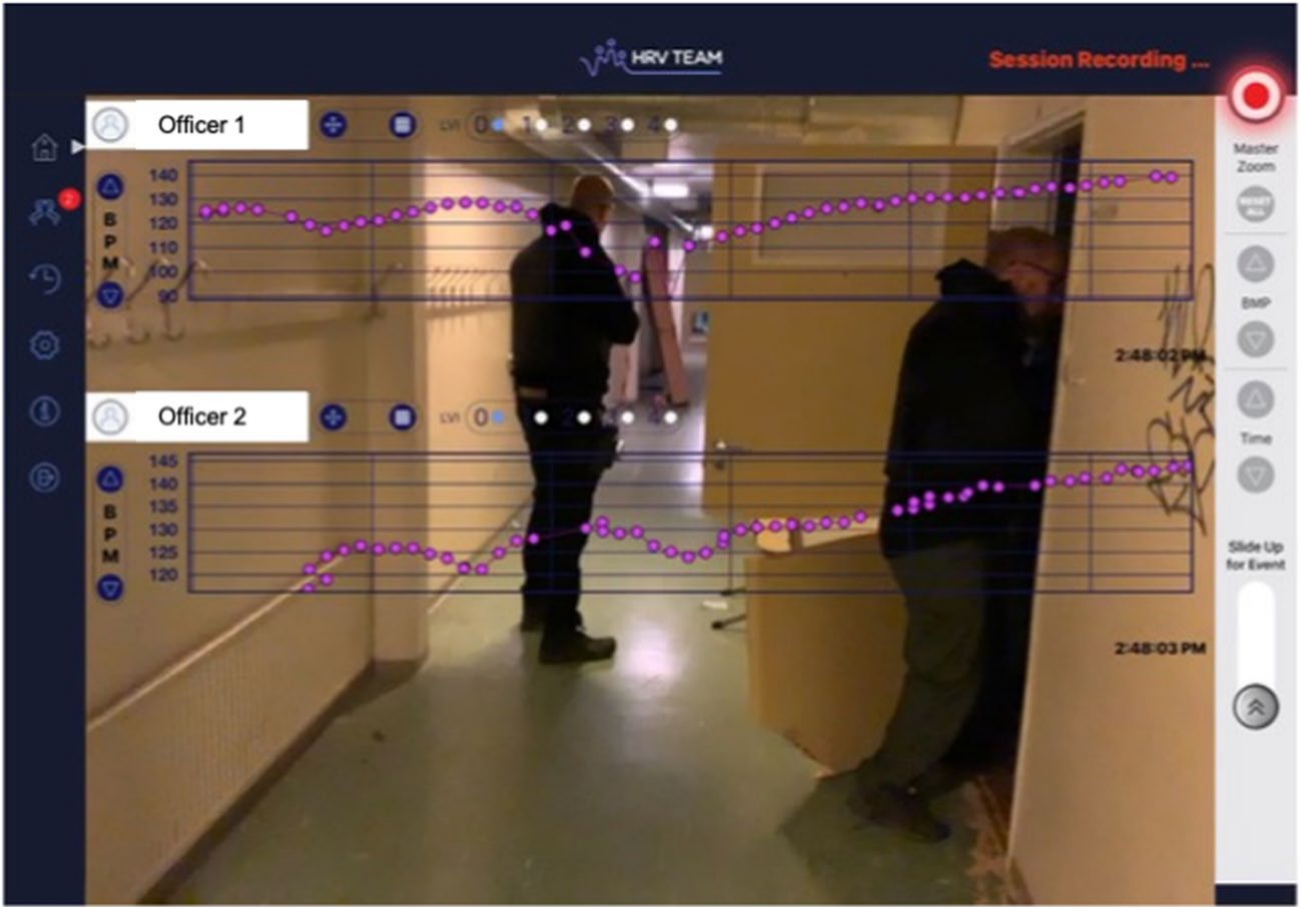
Sample image of HRVB integrated with police RBT. In the HRV Team version of the HRVB app, multiple users’ real-time beat-to-beat heart rate can be visualized (shown by the purple dots plotted in beats per minute on the y-axis and successive beats over time on the x-axis) by configuring the Bluetooth input from multiple wearables. Image provided by H. Gustafsberg

**Fig. 7:**
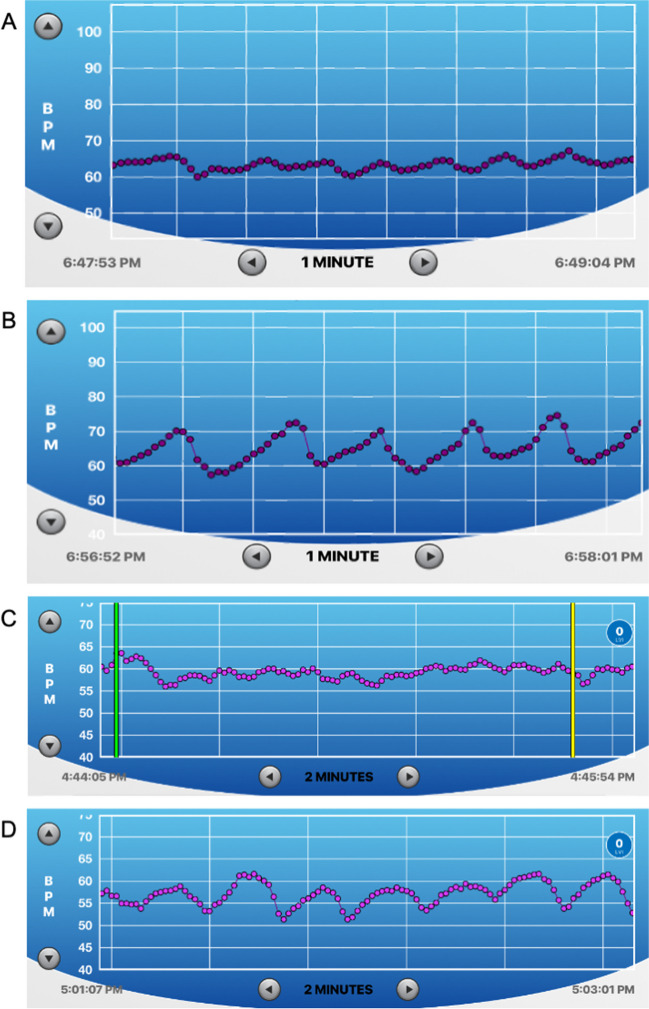
Contrasting heart rate variability during rest and recovery as shown on the HRV Trace app. **a** Continuous heart rate during resting non-paced breathing. **b** Heart rate during paced Recovery Breathing. This individual’s preferred Recovery Breathing pace is 5.5 breaths per minute, which is demonstrated here for 69 s. **c** Heart rate breathing at 7.2 breaths per minute for a duration of approximately 2 min. The green vertical line denotes a start marker in the HRV Trace training mode and the yellow vertical line is an end marker. This is not an optimal Recovery Breathing pace based on poor wave amplitude as indicated in extant HRVB breathing protocols as well as subjective ratings of respiratory discomfort, which previous breathing protocols do not account for (Balban et al., 2023; Lehrer et al., 2013; Steffen et al., 2017; Vaschillo et al., 2006). **d** Heart rate breathing at 4.0 breaths per minute for a duration of approximately 2 min. Visual inspection of the waveform indicates a somewhat smooth and consistent sinusoidal pattern, but inconsistent peak-to-trough amplitude and not comfortable according to the participant. Therefore, this would not be recommended as an optimal Recovery Breathing pace. Data images provided by J.P. Andersen

1. We use the following instructions the first time we attach the HR monitors and connect to the HRVB app (HRV Trace). In this example we use the Polar H10 HR monitors, but any Bluetooth-compatible HR monitor can be used:
  - *We will now be putting on the heart monitors that will transmit your real-time HR to the HRVB app (HRV Trace). Wet the back of the chest strap where indicated and place the strap around your torso, below the sternum and on your skin. Clip in the HR module and pair it to the HRV Trace app in the app itself (not the Bluetooth settings of the iOS device). Pair by tapping the ‘options’ button, click on ‘devices’, and your Polar module device number should be listed. Click the red paperclip icon to the right of your device number and when it turns green, you are connected. Return to the home screen and confirm that your real-time HR is visible moving across the screen, displayed as purple dots* (see Fig. 4 for screen view*).*
2. Officers equip themselves with training and safety equipment (e.g., duty belt, safety vests, modified duty weapon or safe ammunition) and check that their HR monitors are still transmitting to the HRVB app for subsequent post-scenario physiological debrief on RRR.
3. Each officer will engage in a set of three ‘flash scenarios’, also referred to as box drills or reaction drills. Each scenario is designed to be very brief (10–45 s) and induce a fast response from the officer to observe behaviour, state of arousal, tactical skills, situational awareness, and decision-making under pressure in an occupationally relevant task. In most cases, no back story is provided making this a simple ‘reaction drill’ with a clear preferred outcome. These scenarios are not intended to assess complex decision-making skills but rather to elicit physiological stress responses and possibly identify shortcomings in an officer’s reactionary skills with respect to their positioning and equipment (e.g., drawing your flashlight instead of your baton) to direct further training. Each scenario is different and not all of them feature an actor (e.g., clearing rooms) or imminent threat requiring a use of force (for examples contact corresponding author). Multiple scenarios are delivered consecutively to observe the possibility of accumulated stress responses, which has been shown in training and field settings (Andersen et al., 2016a; Bertilsson et al., 2019) and which can impact performance in later scenarios if not addressed with self-regulatory techniques like RRR.
4. Before the first scenario begins, the instructor or an additional facilitator will begin recording the officer using the video function on the HRV Trace app, which superimposes the real-time HRVB trace (Fig. 6). For instructor script pre- and post-scenario see Supplementary Materials.
  - The officer is placed in a physical position where they can’t see the actor enter the environment/training space. The officer could be asked to face a wall or, in some cases, start in the room with their back to the scene. On the instructor’s command, the scenario begins and the officer will turn and be immediately confronted by the actor or scene as relevant to the objective of the RBT. The officer will have only a second or two to make key decisions and respond, as in real life. The instructor will then issue an ‘end scenario’ command.
    i. At the start and stop commands, the facilitator will pull an event marker in the HRV Trace app to manually mark the HRVB data (Fig. 7c).
  - Scenario 1: the participant is not reminded to do RRR because this establishes the baseline physiological stress response captured by the HRVB app.
  - Scenario 2: the officer is coached through the RRR before they begin. Following the scenario, the instructor asks the officer when they could have inserted a Reset breath during the scenario if they did not.
  - Scenario 3: the officer is reminded to do RRR before they begin and insert a Reset Breath during the scenario.
5. Following this last scenario, the participant and trainer review the officer’s real-time HR superimposed on the screen recording of the entire session, which is found in the history log of the HRV Trace. Increases in HR are observed as they correspond to events in the scenario as is seen in Fig. 4 (e.g., suspect behaviours, officer commands or actions). In this way, officers are provided with objective evidence of their physiological arousal, which they are often unaware of either due to social evaluate threat, pressure, or hesitancy to underreport stress (Newell et al., 2022), or simply unaware due to physiological habituation (Chan et al., 2022; Planche et al., 2019). This debrief is the opportunity to 1) objectively view one’s physiological arousal during acutely stressful situations that are representative of the tasks officers face in training and field settings, and 2) use HRVB to condition the adaptive RRR technique.

### Module 4: Intervention for Chronic Stress: Recovery Breathing

The purpose of the last two modules is to introduce participants to an evidence-based intervention that builds wellness reserves and reduces the accumulated health risks associated with chronic stress – **Recovery Breathing**. The current Recovery Breathing protocol is adapted from Lehrer et al.’s (2013) resonance frequency (RF) HRVB breathing protocol but has been modified from its delivery in clinical or laboratory-based settings for operational police contexts using ambulatory equipment. The Recovery Breathing module begins with assisting officers in identifying their individual breathing pace that most closely resembles their optimal RF. Using the HRV Trace app, officers practice breathing briefly at the most common breathing paces (listed below) and visually inspect the HRVB data to identify their Recovery Breathing pace in line with established clinical RF guidelines (Vaschillo et al., 2006).

**Fig. 8:**
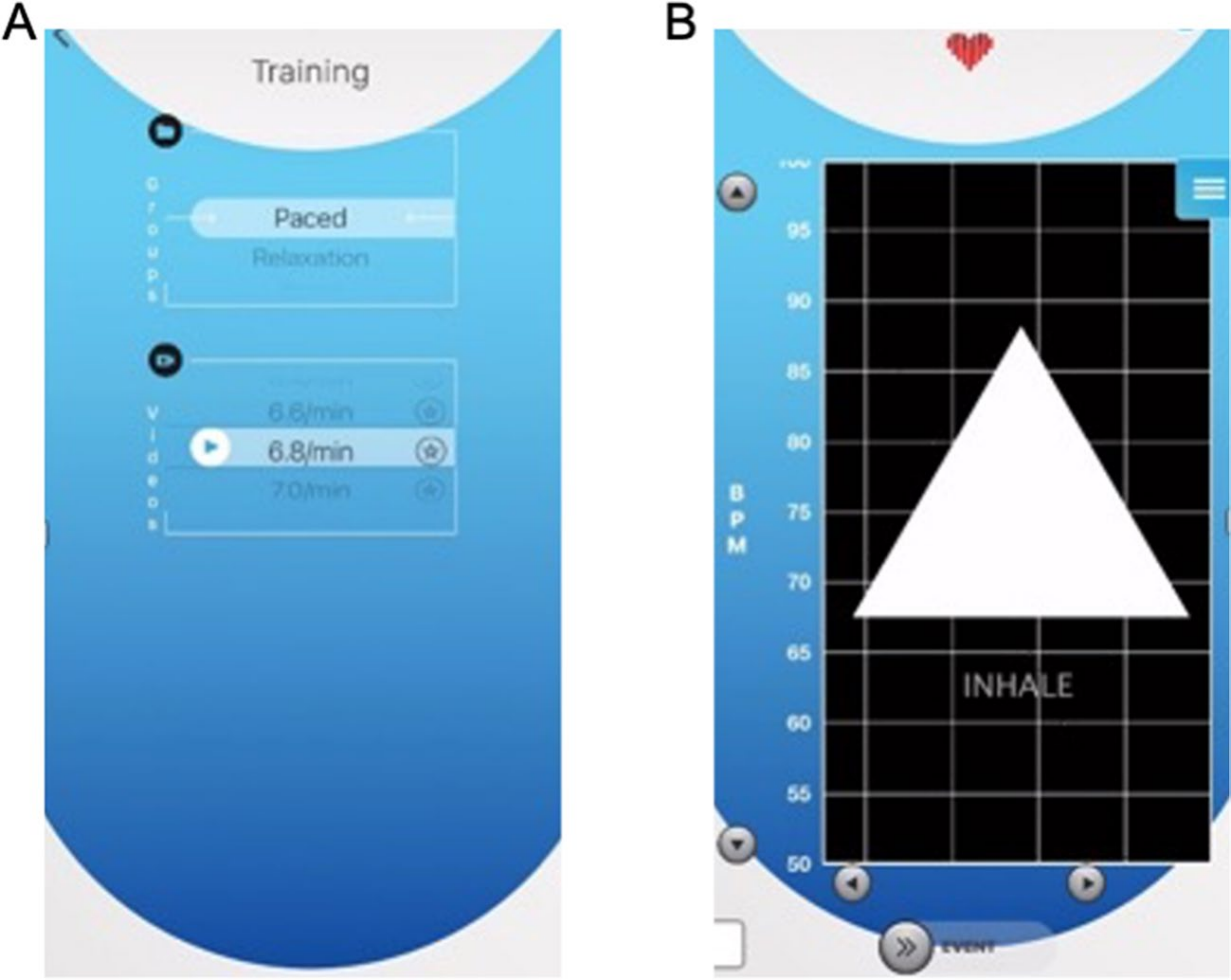
HRV Trace paced breathing training setting. **a** Users can practice breathing at Recovery Breathing paces using audiovisual guides that vary by 0.2 breaths per minute and range from 4.0 to 10.0 breaths per minute. Video training guides can be played by tapping the triangular icon to the left of the pace. Once users identify their own unique Recovery Breathing pace, they can save it as a favorite by tapping the star icon to the right of the pace. **b** Each paced breathing video presents an audiovisual guide indicated by a triangle that grows and the word INHALE presented beneath it in time with the paced inhalation, and shrinks with the word EXHALE presented beneath it in time with the paced exhalation, which is slightly longer than the inhalation duration (2:3 ratio). An auditory prompt also indicates when to “inhale” and “exhale” along in time with the selected pace

1. If not already configured, officers should put on ambulatory HR monitors and connect to the HRV Trace app via Bluetooth connection. Troubleshoot any technical issues prior to beginning the module.
2. Begin with brief instruction on the difference between resting HR, relaxation, and Recovery Breathing because they are not the same physiological states.
  - Periods of time when a person is at their resting HR and ‘relaxing’ like watching television does not actually put them in a state that optimizes vagal tone, oxygen and carbon dioxide exchange in the lungs, and emotional regulation (Lehrer & Gevirtz, 2014). To demonstrate this, we show officers Fig. 7 to contrast resting HR (panel a) from HR during Recovery Breathing (panel b), during which vagal tone is promoted and physiological recovery occurs.
3. “*Every person has a breathing rate which will maximize their optimal level of activation to maintain effectiveness, and this can be seen in the HRVB trace. For adults, the rate is generally between 5–8 breaths per minute, but the exact rate varies from one person to another. The rate is somewhat related to the size of the chest cavity, with larger people typically having a slower recovery breathing rate than smaller people. However, this is only a rough guide. In *Fig. 7b*, we can see a person breathing at his recovery breathing rate. The timestamps at the bottom of the graph show how much time is displayed in the HRVB trace, 69 s. Each wave is one breath and there are about 5 1/2 waves on the screen. So, the person is breathing at about 5.5 breaths per minute. While this is much slower than "normal" the person was quite comfortable. The person was also alert and aware of his surroundings. This "calm and alert" state is one thing that distinguishes recovery from sleep or napping.”*
4. Officers are coached on identifying their own unique Recovery Breathing pace. In a group setting, seven different paces (7.2, 6.8, 6.4, 6.0, 5.6, 5.2) are practiced for two minutes each using the ‘training’ setting in the HRV Trace app (Fig. 8a). The seven paces have been identified in the co-authors collective research, practical, and clinical experience with HRVB in law enforcement populations as the most common paces that capture the majority of the population (Andersen et al., 2018a, 2018b; Arpaia, personal communication).
5. Beginning with the fastest pace (7.2), officers are instructed to follow the audiovisual breathing pacer on their HRVB app (Fig. 8b) while the instructors keep time. The beginning and end of each pace are marked in the HRVB data with green and yellow vertical event markers, respectively (see Fig. 7c above).
  - In time with the pacer, participants are instructed to breathe in through the nose (indicated by the word INHALE and a growing triangle) and out through mouth (indicated by the word EXHALE and a shrinking triangle). They are also instructed not to breathe too deeply or hold their breath in order to avoid ‘over breathing’ (Lehrer et al., 2013). The breath should be smooth, effortless, and comfortable. Officers are also made aware that the exhalation is slightly longer than the inhalation (see Table 1 comparing breathing techniques). The ratio of inhalation time to exhalation time is 2:3. This is subjectively more comfortable for most people and tends to increase parasympathetic activity (Bae et al., 2021). The transition between inhalation and exhalation and vice versa is deliberately imprecise so that the trainee can breathe at a slightly different ratio from breath to breath instead feeling “locked in” to a rhythm. Allowing slight variability in the length of inhalation and exhalation improves comfort and increases the use of the technique in clinical practice (Arpaia, personal communication).
  - If the breathing pace becomes uncomfortable, officers are instructed to stop breathing with the pace and continue breathing at a normal resting rate until any feelings of discomfort subside. If a participant is unable or not comfortable breathing in through their nose (e.g., nose is blocked) they can breathe through the mouth.
6. Following each 2-min practice, officers are instructed to record notes on a handout (see Supplementary Materials) similar to the handout used in Lehrer et al.’s (2013) RF protocol but modified to capture their comfort level (indicated by a Yes/Maybe/No rating) and elements visible on the HRV Trace app (i.e., smoothness of the HR waveform, height of peak-to-trough wave amplitude). Officers are given 1–2 min to breathe as normal before proceeding with the next slower pace.
7. Following the completion of all seven 2-min Recovery Breathing paces, instructors review the HRVB data individually to determine each officer’s ideal Recovery Breathing pace. In accordance with RF HRVB guidelines (Vaschillo et al., 2006), ideal Recovery Breathing paces are identified based on two criteria:
  - Observing a sinusoidal wave pattern in the HRVB data with maximal peak-to-trough amplitude, indicative of synchrony between the heart and lungs and breathing at RF;
  - Self-rated comfort, which is not considered to be of central importance in other paced breathing protocols or investigations (Balban et al., 2023; Lehrer et al., 2013; Steffen et al., 2017; Vaschillo et al., 2006).
  - In the event that an officer does not feel that any of the seven chosen paces are comfortable, an instructor will work with them to find a comfortable pace. The HRV Trace app includes audiovisual training aides for paces ranging by 0.2 breaths per minute from 4.0 to 10.0 breaths per minute.
8. Conclude module with: “*Why is it so important for you to practice Recovery Breathing throughout the day? Recovery Breathing keeps your batteries charged enough to help keep your brain from getting over activated. If you brain is overactivated, then you are much more likely to make mistakes. You are more likely to lose your temper, to lose situational awareness, to miss threat cues that could place you or others in danger. The overactivation can occur even if you do not feel particularly drained. The good news is that 1–2 min of recovery can keep your brain in the safe zone for optimal energy and performance. Let’s look at some real data–the kind you will be seeing on your app.”* For more information on the science of recovery refer to prior literature (Andersen et al., 2023; Andersen et al., 2021; Arpaia & Andersen, 2019).

### Module 5: Integrating HRVB Practice for RRR and Recovery Breathing using RBT

Once participants identify their unique Recovery Breathing pace in Module 4, they are coached on how to practice this technique in Module 5 during acutely stressful RBT that should immediately follow according to the iPREP protocol (see Table S1). The immediate rehearsal of Recovery Breathing, together with the previously trained RRR technique, following each of the RBT scenarios presented in this module promotes learning and skill transfer by way of deliberate practice under conditions functionally equivalent to those in which these techniques will be applied in the field (Ericsson, 2008; Staller et al., 2017).

To maximize limited time for RBT, the following scenarios are typically performed in pairs or teams. Ideally the groups would engage in 3 scenarios (minimum of 2) so that: a) each officer has the opportunity to perform each role (i.e., the primary responder in the RBT, the HRVB debrief coach, and the instructor facilitating the scenario); and b) the Recovery Breathing technique can be used to modulate physiological arousal in preparation for a subsequent scenario or call for service, as recommended for implementation in the field. In accordance with established pedagogical frameworks (Di Nota & Huhta, 2019; Di Nota et al., 2021a, b, c; Jenkins et al., 2020; Körner & Staller, 2020), the following scenarios increase in complexity and physiological arousal relative to the scenarios performed in Module 3 to promote encoding of newly learned RRR and Recovery Breathing skills in representative operational police contexts.

6. Officers equip themselves with training and safety equipment (e.g., duty belt, safety vests, modified duty weapon or safe ammunition) and check that their HR monitors are still transmitting to the HRVB app for subsequent post-scenario physiological debrief on RRR and Recovery Breathing.
7. Prior to the first scenario, officers are provided with necessary briefing information, coached with a Reset Breath and grounding to get into a ready mindset, and reminded to implement a Reset Breath wherever they feel it would be natural or useful in the scenario.
  - At the start and end of the scenario (i.e., when the instructor blows a whistle), the instructor, assistant, or officer holding the HRVB app should swipe an event marker to identify the beginning/end of the scenario in the recorded session data.
8. Immediately following the scenario, officers are coached on performing a Reset Breath in order to reduce physiological arousal induced by the RBT and achieve an optimal physical and mental state that is receptive to learning and knowledge retention (Fig. 3c, Di Nota & Huhta, 2019).
9. Officers are individually debriefed by an instructor (or peer) on their performance (captured by the HRV Trace video function) and superimposed real-time HRV trace (see Fig. 6).
  - Review the scenario HRVB session data and draw officers’ attention to increases in HR that are typically observed prior to the start of the scenario (i.e., anticipatory stress) and which continue to increase as the scenario progresses. Any physiological evidence of a Reset Breath (i.e., significant decrease in HR) are noted in the continuous HRVB data and discussed in accordance with scenario performance. Trainers are taught to distinguish decreases in HR due to a Reset Breath from similar changes in HR due to significant changes in posture (e.g., quickly bending forward to touch toes also resembles a reset breath).
  - Officers are asked to reflect on their physical sensations before, during, and after the scenario, applicability of the Reset Breath in the scenario and in future implementation.
10. Following the HRVB debrief, officers are instructed to sit alone in a separate room or area from the RBT and practice 5-min of Recovery Breathing at their preferred pace using the HRV Trace training aids (Fig. 8). This period allows for consolidation of the newly conditioned Recovery Breathing pace following an acutely stressful event. As training progresses, officers are encouraged to gradually reduce reliance on visually following the pacer and just doing the breathing naturally, only checking the HRVB trace/pacer periodically to see if they remain on pace. By doing so, their Recovery Breathing pace will be conditioned *without relying on external technological aids.* Behavioral conditioning one’s breathing pace is possible and essential, as it is not recommended to use external technological aids during field duty. Rather, technological aids are intended for training only and accelerate encoding and generalization.

## Technological Developments for HRVB in Field Settings

A quick search on any app platform shows that there are numerous HRVB apps and software systems commercially available for sports or stress and health monitoring. There are several key reasons our iPREP team (JA) developed a customized suite of apps (HRV Train; HRV Trace; HRV Team) designed for use in clinical settings that also possess several advantages for use in field settings by active-duty law enforcement officers. The HRV Trace app can simultaneously record HRVB and video, HRV Train does not have the video function, and HRV Team can record video and simultaneous HRVB from multiple users/wearable inputs. These HRVB apps are only available on the Apple iOS platform at the time of this publication but are Bluetooth-compatible with a wide range of commercially available HR monitors, including Polar and Zephyr.

Advantages of using the bespoke suite of HRVB apps described in this paper include:

1. Security and privacy are paramount when measuring physiological signals and police behavior and training. For that reason, all our HRVB applications securely store deidentified physiological data locally on the device (i.e., iOS smartphone or tablet) and it is never uploaded to a cloud-based server or ever shared with third parties (for further information on app security see Andersen et al., 2023). Almost all commercial apps reveal or sell data to third party entities and cannot guarantee privacy for protecting your trainee’s data.
2. Our HRVB suite of apps can be used with HR monitors that record beat-to-beat data to measure cardiovascular parameters (i.e., R-R intervals) that better approximate HRV using industry standard software (Kubios). Furthermore, these apps visualize the immediate (i.e., **with no time delay**) and raw HR signal (i.e., no composite scores or manipulation by proprietary algorithms) for ease of interpretation by end users. The app interfaces and features have also been custom designed for police with simplified display screens, video recording capabilities to capture HRVB during active RBT (Fig. 6), and event markers that mark physiological data for detailed and straightforward analyses (Fig. 7c).
3. Heart rate variability is the beat-to-beat variation in the heart period (R-R interval) or the heart rate (HR). This beat-to-beat variability can be summarized by various HRV metrics such as high frequency (HF), low frequency (LF) HF/LF ratio, and root mean square of successive differences between normal heartbeats (RMSSD), which are often used synonymously with HRV. However, any individual HRV metric is a summary of the beat-to-beat variability over time. Many commercially available apps, devices or programs claim that particular HRV metrics, or combinations thereof, are more sensitive indicators of stress or health upon which they design an algorithm that produces an index of ‘stress’ or ‘coherence’ that is even further removed from the raw HR signal. What is often not explained or understood is that it is HR that is responding to the acute neural activation or inhibition of the ANS (Billman, 2013) and thus is the variable to be modified through direct observation and measurement in ambulatory HRVB training. While HRV metrics may be more sensitive indicators of stress or health, they are also highly non-specific and are influenced by factors such as respiration rate and depth, posture and movement, time of day, food or water consumption, bladder filling, or phase of the menstrual cycle (see Heathers, 2014; Quintana & Heathers, 2014). Thus, relying on HRV metrics or associated algorithms and indices like a ‘stress index’ for behavioral modification inherently produces misleading or non-interpretable results because it is impossible to determine what portion of the HRV metric truly represents cardiac autonomic and neural signals due to the variables of interest, and what is due to noise or unrelated physiological input. Given these scientific principles, we caution the researchers and practitioners designing programs for police that use proprietary algorithms of HRV ‘stress’ indicators to predict health or performance outcomes, as they may be misleading and result in either no benefit or even detrimental effects on participants’ health or performance.

## Application of Controlled Breathing Techniques in Active Law Enforcement Settings

Law enforcement and military populations commonly utilize various breathing techniques to optimize performance, recovery, and health (see Table 1 for summary). Perhaps the most popular breathing technique is called tactical (or combat or box) breathing, characterized by a person counting equal duration of inhalation, hold, exhalation, and hold again for 4 or 5 s each. Use of this breathing practice is reported in the lived experience of military operatives (Grossman, 2014). However, empirical studies of tactical (or combat or box) with military cadets revealed that it was associated with reduced performance (i.e., accuracy and reaction time during experimental tasks) compared to prolonged exhalation (Röttger et al., 2021). These findings are unsurprising given that holding one’s breath limits necessary oxygen intake and CO_2_ gas exchange (which may stimulate hyperventilation). Further, the process of counting 1–4 during each phase (inhale, hold, exhale, hold) is distracting from the operational task at hand because it requires cognitive resources that are already limited during physiologically stressful police contexts (Kleygrewe et al., 2023).

Most recently, cyclic sighing has been promoted in popular media as an adaptive breathing technique in active law enforcement settings (e.g., following reality-based scenarios or service calls). However, the only empirical investigation of cyclic sighing was an online study with no participant description (i.e., not a confirmed law enforcement sample) where adherence and correct performance could not be verified, and more critically was not tested for effectiveness in operational police settings (Balban et al., 2023). The psychophysiological model of sighing (i.e., prolonged exhalation) demonstrates that this technique *when performed in isolation* (i.e., as a single sigh) can reset dysregulated respiratory cycles by promoting vagal control when sympathetic arousal is high, inducing a sense of relief similar to the Reset Breath. However, respiratory scientists caution against the excessive use of repeated sighing as it may contribute to an escalating cycle of respiratory instability and induce feelings of subjective discomfort (Vlemincx et al., 2013). A concern with using cyclic or repeated sighing during operational police tasks is that it shifts focus to the physiological discomfort and unease, rather than directing attention to ‘what’s is important now.’

Taken together, this evidence highlights a potential danger of introducing breathing techniques that are mismatched with the operational demands present in active law enforcement settings. Non adaptive or untested breathing experiments may lead to potential deficits in respiratory control, self-regulation, performance, occupational health, and safety of both operators and the public. We urge police and law enforcement officials to evaluate: 1) the applicability of evidence for operational contexts before endorsing or training officers in any controlled breathing techniques, and 2) The validity of claims made by emerging or commercial entities that lack or mischaracterize the evidence supporting their products or training. As decades of research have shown in applied and clinical research, there is no ‘one size fits all’ solution to adaptive stress modulation. Rather, evidence-based training must be tailored for, and tested among, representative police samples to observe immediate and prolonged effects on health, safety and performance.

## Conclusions

While the current HRVB protocol was developed to address the occupational demands and stressors specific to law enforcement and tactical intervention teams, the intervention techniques and underlying physiological mechanisms bear relevance to professionals in other high-stress occupations, including public safety and frontline healthcare workers, and in non-operational contexts (e.g., during court testimony, administrative or organizational contexts, see Di Nota et al., 2020).

The current HRVB protocol also accounts for challenges and limitations imposed by the realities of police work, including the prioritization of operational duties over participation in training and research, both of which are above and beyond mandated training requirements. Our studies have tried to integrate HRVB training into regular RBT or evaluation, including annual requalification evaluations, in order to maximize participation while minimizing the burden placed on participating agencies (i.e., time, funding, personnel, infrastructure, equipment) (Andersen et al., 2018a, b; Di Nota et al., 2021b). Seamless integration of the current HRVB protocol and larger iPREP programs has enabled us to recruit large participant samples that are nonetheless prone to attrition when requested to attend follow-up evaluations due to relocation, change of schedules, or loss of interest.

As a result of the demonstrated efficacy of the current protocol in improving performance (situational awareness, lethal force decision-making) and physiological (post-scenario HR recovery) outcomes, iPREP has been integrated into standard police training at the national level in Finland and Iceland and broadly trained across a number of agencies in North America (see Andersen et al., 2021). Since 2017, iPREP has been accredited as a Continuing Professional Development course by the University of Toronto Temerty Faculty of Medicine, from which participants receive a Continuing Professional Development credit following course completion. We have also adapted a Train the Trainers (TT) version of iPREP to facilitate broad knowledge translation and education among law enforcement agencies. Most recently, the lead author has received support from a Canadian federal priority research grant in public safety wellness to adapt the current HRVB protocol for online remote delivery (Andersen et al., 2023). The Autonomic Modulation Training (AMT) intervention is intended to reach police practitioners in remote detachments that may not have the resources or access to evidence-based training that supports health, wellness, and performance.

### Supplementary Information

Below is the link to the electronic supplementary material.

Supplementary file1 (DOCX 15 KB)

## Data Availability

To protect the privacy and confidentiality of participants, which are comprised of active-duty police officers, the data sets generated and/or analyzed over the course of this research program are not publicly available. De-identified and/or aggregate data may be available from the principal investigator (JPA) on reasonable request.

## Abbreviations

*AMT*: Autonomic Modulation Training
*ANS*: Autonomic nervous system
*BPM*: Beats per minute
*HF*: High frequency
*HR*: Heart rate
*HRV*: Heart rate variability
*HRVB*: Heart rate variability biofeedback
*iPREP*: International Performance Resilience and Efficiency Program
*LF*: Low frequency
*PNS*: Parasympathetic nervous system
*PSP*: Public safety personnel
*RF*: Resonance frequency
*RMSSD*: Root mean square of successive differences between normal heartbeats
*RRR*: Reset, Refocus, Respond
*RBT*: Reality-based training
*RSA*: Respiratory sinus arrythmia
*SNS*: Sympathetic nervous system
*SWAT*: Special weapons and tactics
*TT*: Train the Trainers
