# Supplementary material for: The International Performance, Resilience and Efficiency Program Protocol for the Application of HRV Biofeedback in Applied Law Enforcement Settings
Source: Appl Psychophysiol Biofeedback. 2024 Apr 24;49(3):483–502. doi: 10.1007/s10484-024-09644-3 (PMC11310253; doi:10.1007/s10484-024-09644-3)
Supplement: Supplementary file 1 — Supplementary file1 (DOCX 15 KB) [file 10484_2024_9644_MOESM1_ESM.docx]

**Supplementary Materials**

**Table S1.** Suggested HRVB training schedule

| Schedule | Day 1 | Day 2 |
| --- | --- | --- |
| Morning | Module 1: Background and Intro to Stress Physiology and HRVB  Module 2: Intervention for Acute Stress: RRR | Module 4: Intervention for Chronic Stress: Recovery Breathing |
| Afternoon | Module 3: Practice RRR using HRVB and RBT | Module 5: Integrating HRVB Practice for RRR and Recovery Breathing using RBT |

### *Note:* Additional days of HRVB and RBT integration can be added as needed for training or research purposes. (see Andersen & Gustafsberg, 2016; Andersen et al., 2015; 2016; 2016a; 2018). HRVB – heart rate variability biofeedback; RBT- reality based training; RRR – Reset, Refocus, Respond technique. Of note, the iPREP Train the Trainer’s course (not described in full in this paper) is a three day course taught by Master iPREP Trainers. For more information on the content of the full TTT course, please contact the lead author (J.P. Andersen).
